# Regional Spondylodiscitis Disparities: Impact on Pathogen Spectrum and Patients

**DOI:** 10.3390/jcm13092557

**Published:** 2024-04-26

**Authors:** Tobias Pantel, Klaus Christian Mende, Martin Stangenberg, Malte Mohme, Theresa Mohme, Frank Floeth, Sven Oliver Eicker, Marc Dreimann

**Affiliations:** 1 Department of Neurosurgery, Hamburg University Medical Center, Martinistrasse 52, 20246 Hamburg, Germany; 2 Department of Neurosurgery, Friedrich-Ebert-Krankenhaus, Friesenstr. 11, 24534 Neumünster, Germany; 3 Department of Trauma and Orthopedic Surgery, Hamburg University Medical Center, Martinistrasse 52, 20246 Hamburg, Germany; 4 Department of Spine and Neurosurgery, Tabea Krankenhaus Hamburg, Kösterbergstraße 32, 22587 Hamburg, Germany; 5 Wirbelwerk Hamburg, Orchideenstieg 12, 22297 Hamburg, Germany; 6 Department of Spinal Surgery, Hospital zum Heiligen Geist, Von-Broichhausen-Allee 1, 47906 Kempen, Germany; 7 Department of Spine and Scoliosis Surgery, Lubinus Clinicum, Steenbeker Weg 25, 24106 Kiel, Germany; 8 Department of Spine, Orthopädische Klinik Markgröningen, Kurt-Lindemann-Weg 10, 71706 Markgröningen, Germany

**Keywords:** spinal infection, antibiotic therapy, immunosuppressive conditions, multidisciplinary therapy outcome, concomitant malignancy

## Abstract

**Background**: Spondylodiscitis is an infectious disease affecting an intervertebral disc and the adjacent vertebral bodies and is often the complication of a distant focus of infection. This study aims to ascertain the regional and hospital-specific disparities in bacterial patterns and resistance profiles in spontaneous and iatrogenic spondylodiscitis and their implications for patient treatment. **Methods:** We enrolled patients from two German hospitals, specifically comparing a university hospital (UVH) with a peripheral non-university hospital (NUH). We documented patient demographics, laboratory results, and surgical interventions. Microbiological assessments, antibiotic regimens, treatment durations, and resistance profiles were recorded. **Results:** This study included 135 patients. Upon admission, 92.4% reported pain, with 16.2% also presenting neurological deficits. The primary microbial species identified in both the UVH and NUH cohorts were *S. aureus* (37.3% vs. 31.3%) and cog. neg. staphylococci (28.8% vs. 34.4%), respectively. Notably, a higher prevalence of resistant bacteria was noted in the UVH group (*p* < 0.001). Additionally, concomitant malignancies were significantly more prevalent in the UVH cohort. **Conclusion:** Significant regional variations exist in bacterial prevalence and resistance profiles. Consequently, treatment protocols need to consider these nuances and undergo regular critical evaluation. Moreover, patients with concurrent malignancies face an elevated risk of spondylodiscitis.

## 1. Introduction

Spondylodiscitis is a serious medical condition characterized by the infection of an intervertebral disc and the adjunct vertebral bodies. Its prevalence in Western countries is estimated to range from 4 to 24 million cases annually, with an increasing trend [1,2,3].

Patients with spondylodiscitis may exhibit a diverse spectrum of clinical symptoms, and the severity of the infection can vary significantly [4,5,6]. It is not uncommon for these patients to exhibit overlapping symptoms with the primary infection. Essentially, symptoms may vary from isolated back pain, which typically worsens within the movement, to acute paraplegia caused by a space-occupying intraspinal abscess or a collapsed vertebral body [7,8].

The majority of spondylodiscitis cases arise as complications of a distant infectious focus disseminated either through hematogenous seeding or continuous spread from the primary site of infection [3,5]. Moreover, spondylodiscitis may occur iatrogenic as a consequence of infiltration treatments or spinal surgery. The primary infection’s focus remains unidentified in nearly 50% of all cases [7]. Patients with a weakened immune system, for example, due to metabolic syndrome or immunodeficiency syndrome, are particularly at risk [9].

Timely identification and appropriate intervention are imperative to minimize the associated morbidity and mortality [10,11]. However, the diagnosis of spondylodiscitis is frequently delayed due to the occasionally nonspecific clinical symptoms. The main course of treatment is conservative administration of antibiotics [12] and sanitation of the primary septic focus [11,13]. Nevertheless, surgical intervention is often necessary to obtain intraoperative bacterial specimens, manage complications such as instability or abscess formation, and perform primary decompression of the spinal infectious focus, particularly in patients with neurological deficits [12,13]. Even with modern treatment approaches, there is a high reported mortality ranging from 2 to 20% [2,14,15].

Regarding the microbial characteristics of spondylodiscitis, it is increasingly recognized that the bacterial composition varies regionally. This is recognized and understood in the context of the basic categorization of spondylodiscitis into specific etiologies, such as spondylodiscitis caused by fungi, Mycobacterium tuberculosis or Brucella, and non-specific pyogenic spondylodiscitis, which is prevalent in Europe and North America [7,8]. The latter, in turn, is mainly caused by *Staphylococcus* aureus, *Streptococcus* species, *Pseudomonas aeruginosa*, and *Enterococcus* species [8,16,17,18]. Due to several factors, the specific variant of spondylodiscitis has gained importance in clinical practice in Europe and North America in recent years, further emphasizing the influence of geographical factors on this disease [13]. Ultimately, the spectrum of pathogens causing non-specific spondylodiscitis can vary regionally and includes both supra-regional and local variations [19]. In line with this, Fritzenwanker et al. found regional differences in the distribution patterns of carbapenem-resistant pathogens, which also contribute to spondylodiscitis, in a study [20]. Furthermore, Namvar et al. describe the increasing importance of coagulase-negative Staphylococcus epidermidis as a classic hospital-acquired pathogen that is increasingly emerging as a cause of spondylodiscitis in an aging and medically compromised population [21]. Due to its biofilm-forming capability, this factor must be considered in the treatment of spondylodiscitis, especially when surgical intervention with dorsal stabilization is required [21]. It can be assumed that similar regional differences exist for other pathogens, although this has not been clearly demonstrated in studies.

Based on this, the objective of this study was to assess regional and hospital-specific disparities in microbiological findings, focusing on bacterial spectrum and resistance patterns, and to analyze their implications on the efficacy of patient treatment.

## 2. Materials and Methods

The study was reported to the responsible Ethics Committees (local ethical review board of Hamburg, Germany (WF-013/20, date: 28 January 2020) and was performed in accordance with the ethical standards laid down in the Declaration of Helsinki and its later amendments. We identified patients from two German hospitals, a university hospital (UVH) and a peripheral non-university hospital (NUH), who were treated for spondylodiscitis within a 24-month period (2013–2014).

UVH is a level 1 spine center, according to the German Spine Society in Hamburg, Germany. It offers comprehensive spinal surgery services on a 24/7 basis and performs around 2000 surgical procedures per year. The NUH, located in Kempen, North Rhine-Westphalia, Germany, is a level 3 spine center certified by the German Spine Society. This certification confirms that a wide range of spinal procedures are performed. The annual number of cases at the NUH is around 900 operations.

Patients from both departments who were older than (1) 18 years and (2) diagnosed with spondylodiscitis of any type were included in this study. Patient- and disease-specific parameters were recorded. These included patient demographics (e.g., age, sex), inpatient stay data (length of stay in the normal ward as well as intensive care unit (ICU)), and initial symptoms as well as neurological dysfunctions. In addition, various co-morbidities (e.g., diabetes mellitus, renal insufficiency, intravenous drug abuse, concomitant malignant diseases) were recorded. Laboratory parameters, C-reactive protein (CRP), and leukocyte counts were recorded for the days of admission, surgery, and discharge. Further, the type of surgical treatment and number of surgeries were recorded. Microbiological workup was included if available. Microbes were grouped into *S. aureus*/coagulase-negative staphylococci (CNS)/*Enterobacterales*/*Streptococcus* sp./*Enterococcus* sp./*Mycobacteriaceae*/miscellaneous species. Also, antibiotic treatment, duration, and antibiotic resistance were recorded for analysis.

### Statistics

Data are displayed as mean ± standard deviation (sd) for continuous variables or absolute (n) and relative (%) numbers for categorical variables. Analysis was performed using SPSS Inc. (Version 25, IBM, Chicago, IL, USA), using the Chi^2^ test and student’s *t*-test. A *p*-value of <0.05 was deemed significant.

## 3. Results

### 3.1. Admission and Demographic Parameters

During a 24-month period, we enrolled 135 patients treated for spondylodiscitis, with 68 treated at UVH and 67 at NUH. Of these patients, 51 (37.8%) were female, with a mean age of 66.4 ± 14.4 years (range 19–93 years). Among them, 64 patients (53.3%) were referred with spondylodiscitis, and upon admission, 92.4% (n = 109) experienced pain, while 16.1% (n = 19) exhibited neurological deficits. Myelopathy was present in 10.3% (n = 12) of patients, and 6.0% (n = 7) presented with radiculopathy. Infectious symptoms such as fever were reported in 11.6% (n = 15) of cases, and leukocytosis was observed in 41.9% (n = 54), whereas C-reactive protein elevation was a common finding in 94.8% (n = 127) of patients. Previous spinal operations were reported in 17.8% (n = 24) of cases, with an equal distribution between UVH and NUH. Similarly, previous spinal infiltration therapy, suspected as the cause of infection, was reported in 7.4% (n = 10) of cases, evenly distributed between both cohorts. A statistically significant difference was noted for the presence of malignancy (30.9% (n = 21/68) vs. 9.0% (n = 6/67, *p* < 0.001)). Interestingly, no statistically significant difference was observed for diabetes, purulent infections, intravenous drug abuse, and renal insufficiency (Table 1 and Table 2).

### 3.2. Surgical Treatment

**Figure 1:**
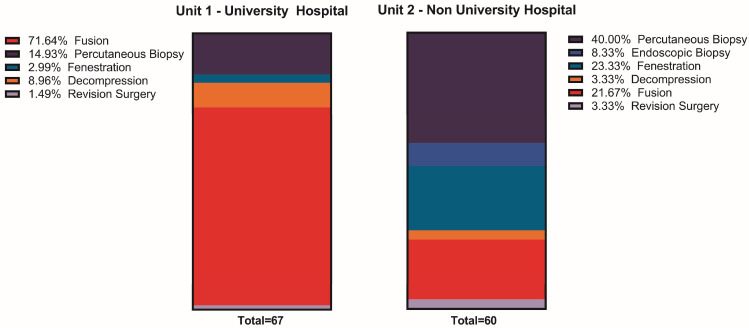
Comparison of initial surgical treatment between UVH and NUH. Chi^2^ *p* < 0.001.

Surgical intervention was necessary for 98.5% (n = 67/68) of UVH patients and 89.6% (n = 60/67) of NUH patients. A higher proportion of UVH patients required multiple surgeries compared to NUH patients (41.1% vs. 19.4%, *p* < 0.002). Percutaneous biopsies were the primary treatment modality for NUH patients (40.0%, n = 24/60), followed by decompression (26.6%, n = 16/60), whereas spinal fusion was predominant at the UVH center (71.6%, n = 48/67) along with percutaneous biopsy (14.9%, n = 10/67, *p* < 0.001). Among patients requiring a second surgery, additional spinal fusion was performed in 53.6% (n = 15/28) at the UVH center compared to 14.3% (n = 2/14, *p* = 0.016) at the NUH (Figure 1).

### 3.3. Microbiological Parameters

**Figure 2:**
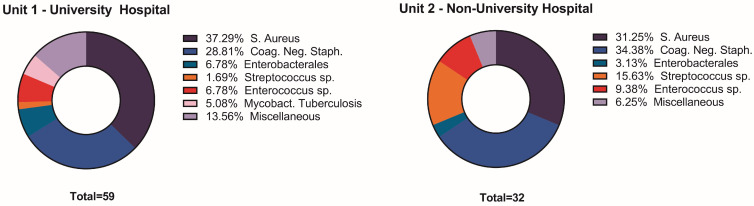
Bacterial specimen by Hospital UVH vs. NUH. Miscellaneous and tuberculosis bacteria were reported more frequently at the UVH; however, they did not reach statistical significance. Chi^2^ *p* = 0.13 (NS).

The predominant microbial species at the UVH were *S. aureus* (37.3%; n = 22/59) and cog. neg. staphylococci 28.8% (n = 17/59) as well as at the NUH center coag. neg. staphylococci (34.4%; n = 11/32) and *S. aureus* (31.3%; n = 10/32). Mycobacteria were detected in 5.1% of UVH patients (n = 3/59), and “other” bacteria were detected more frequently in UVH patients (13.6%, n = 8/59) than in NUH patients. However, statistical significance failed (6.3%; n = 2/32, *p* = 0.13, Table 3, Figure 2). Among the patients included in this study, multiple pathogens were microbiologically detected in 9 cases (6.7%). Significantly, a higher incidence was observed among patients treated at UVH compared to NUH (UVH: n = 8 vs. NUH: n = 1; *p* < 0.001).

### 3.4. Antibiotic Treatment

**Figure 3:**
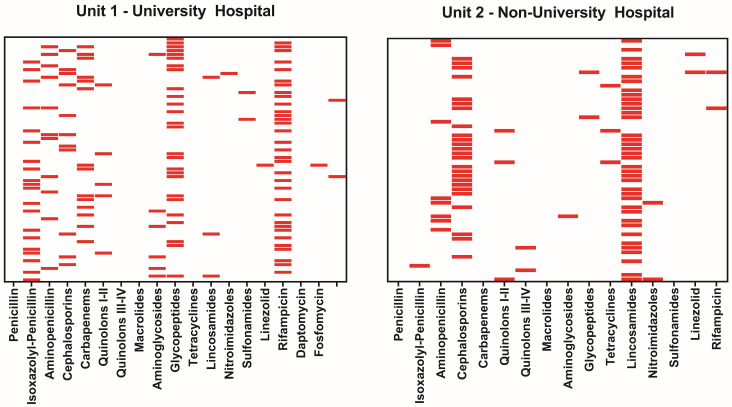
Antibiotics used by the center. The red bar indicates the antibiotic was used, one row per case.

Antibiotic treatment was administered intravenously for an average of 29.6 ± 22.9 days at UVH and 9.8 ± 4.4 days at NUH (*p* < 0.001). The total duration of therapy averaged 7.2 ± 5.9 weeks in UVH and 12.2 ± 3.6 weeks in NUH (*p* < 0.001). Empirical initial antibiotic treatments were more prevalent in NUH, with 64.2% (n = 43/67) compared to 33.8% (n = 23/68, *p* < 0.001) in UVH. The most frequently used combinations at UVH were rifampicin and flucloxacillin (isoxazolyl penicillin) in 16.2% and rifampicin and vancomycin (glycopeptide) in 13.2%. In NUH, the combination of cefuroxime (cephalosporin) and clindamycin (lincosamide) was preferred in 29.9%, followed by clindamycin monotherapy in 16.4% (Table 3 and Table 4, Figure 3). The 9 patients with multiple germ detections received intravenous antibiotics for an average of 31 ± 24.9 days at UVH and for 14 days at NUH.

### 3.5. Resistances

**Figure 4:**
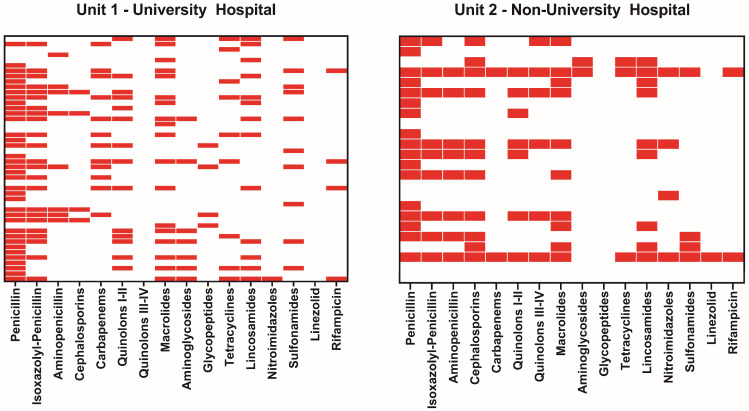
Antibiotic resistances by center. The red bar indicates resistant species. One row per case. Cases with missing information regarding resistance were excluded from this analysis.

Resistance data were available for 46 UVH patients and 24 NUH patients. High rates of resistance were observed to classical penicillins (70.8–78.3%) and isoxazolyl penicillins (37.5–50.0%), as well as to lincosamides (28.3–37.5%) and macrolides (39.1–41.7%). Resistance to cephalosporins was 8.7% in UVH compared to 45.8% in NUH (*p* < 0.001). In addition, resistance to carbapenems was detected significantly more frequently at UVH (n = 12 vs. n = 2 (NUH); *p* < 0.05), Figure 4). When examining the administered antibiotics, a notable proportion of empiric antibiotics were prescribed against resistant species; for example, 50% of aminoglycosides (n = 2/4) and 66.7% of lincosamides (n = 2/3) used in the UVH center (Table 4, Figure 4). Regarding patients with multiple detected pathogens, resistance patterns to more than three antibiotic groups were identified in 6 cases (66.7%).

## 4. Discussion

In this study, we investigated spondylodiscitis patients from two geographically independent spine centers with special attention to the microbiological characteristics of the two cohorts, focusing in particular on the microbiological spectrum and resistance patterns. The main findings of our study are the following: (1) The prevalence of the two predominant pathogens (*S. aureus* and cog. neg. staphylococci) varied between the two spine centers. (2) Resistant pathogens were significantly more common in the University Hospital (UVH), which matches the (3) substantial differences in resistance profiles between the two spine centers. In addition, our data suggest that (4) patients with malignancies have an increased risk of spondylitis.

Evidence supporting the treatment of pyogenic spondylodiscitis remains limited; despite the existence of clinical recommendations [10,13,22,23,24], data validation through controlled trials is scarce [23]. The results of this study reveal a substantial degree of heterogeneity between the two centers regarding patient demographics, disease characteristics, and spondylodiscitis treatment approaches. These disparities probably originate from variations in patient populations and the local bacterial profiles at each center, necessitating individualized treatment strategies. Locoregional differences in patient cohorts can pose distinct challenges and comorbidities, as illustrated by the University Hospital (UVH) managing a significant proportion of pyogenic spondylodiscitis cases in patients with concomitant malignant diseases (30.9%) compared to the NUH (9.0%, *p* < 0.001). This finding is particularly interesting in context. Commonly recognized risk factors for spondylodiscitis, such as diabetes mellitus, immunodeficiency syndrome, renal insufficiency, and intravenous drug use, have been extensively documented in the literature and confirmed by clinical experience. However, our analysis yielded unexpected results; statistically significant prevalence of these diseases in the patient cohorts was lacking, except for concomitant malignancy, as previously noted. Comparable data on this observation are scarce in the existing literature, with only limited studies available to date [25]. An apparent rationale for the elevated occurrence of malignant diseases in this patient cohort likely stems from the compromised immune system associated with the malignancy itself, further exacerbated by corresponding therapies, even though no significant levels were reached for chemotherapy (Table 2). The notably higher incidence of these concomitant malignant diseases in the UVH cohort can be primarily attributed to the hospital’s high-volume oncology center, which has regional significance and, consequently, a larger patient volume. In summary, it is essential to consider spondylodiscitis as a potential diagnosis in patients with malignant diseases, nonspecific infection situations, and back pain, alongside other differential diagnoses.

Next, we want to turn our attention to the microbiological aspects. Our data indicate that uncommon bacteria (excluding staphylococci/enterobacteria, streptococci) exhibited a significantly higher prevalence in patients treated at UVH for spondylodiscitis (18.7% (UVH) vs. 6.3% (NUH); *p* < 0.05). The exact reasons for this disparity are certainly manifold. One possible explanation can be found in the different geographical localization of the departments; the impact of this factor on the bacterial spectrum was documented by Hatsuda et al. [26]. Further, previous treatments in patients with higher morbidity levels, leading to a greater number of hospital-acquired bacterial specimens in these individuals, could contribute to this finding [27]. While a statistically significant difference was not observed, there was a disparity in the bacterial spectrum between the UVH and NUH concerning the predominant pathogens, *S. aureus,* and cog. neg. staphylococci. *S. aureus* was the most commonly identified pathogen at the UVH, whereas its detection rate was lower at the NUH, with a cog. neg. staphylococci are more frequently detected there. This suggests a regional variation. Considering the findings of Cheung et al. regarding the pathogenicity of *S. aureus* and Namvar et al. on S. epidermidis, this disparity gains significance, as both pathogens exhibit distinct pathogenic behaviors, may affect different patient demographics, and consequently, the disease course could vary significantly [21,28].

Bacterial resistances were quite different, with significantly more resistances to Nitroimidazoles (16.7% vs. 2.2%, *p* < 0.05), Quinolons Type III/IV (25.0% vs. 0.0%, *p* < 0.001) and Cephalosporins in the NUH setting (45.8% vs. 8.7%, *p* < 0.001), a phenomenon which may also be related to local bacterial resistance patterns, however especially cephalosporins are a widely used antibiotic agent and were expected to have higher rates of resistances in both hospitals [19,29]. The reason why UVH patients had <10% resistance to cephalosporins remains unanswered as this contradicts all expectations; a selection bias in this still-small sample might be a viable explanation.

In general, we observed a high rate of resistance to Isoxazolyl penicillins (UVH: 50.0%, NUH: 37.5%), which were empirically administered at UVH. Since they were commonly combined with Rifampicin (8.7% resistant), no specimens in this cohort were resistant to both antibiotics. Nevertheless, a 50% resistance rate in an empiric antibiotic regimen is unacceptably high and warrants a reevaluation of the agents used. The same applies to the NUH center; the two antibiotics administered in combination as an empiric treatment regimen exhibited equally high resistance rates of 45.8% in Cephalosporins and 37.7% in Lincosamides. The legitimate question is whether these resistances have been prevalent for some time and empiric regimens were simply inadequately chosen or if regional resistance patterns have changed over time, as demonstrated, for instance, by the CANWARD Study [30]. Another remarkable finding from our analysis is the significant increase in carbapenem resistance in the UVH cohort. Fritzenwanker et al. highlighted this escalating resistance problem in a 2018 review [20]. While these resistance patterns were initially prevalent in south-eastern Europe, there is now evidence of increasing prevalence in Germany, which is consistent with the results presented here. The significant deviation between the two departments investigated underlines the existence of regional disparities and once again highlights the need to recognize and take into account regional characteristics in the pathogen spectrum. The microbiological findings described influenced the success of treatment in both centers. The use of empirical antibiotics in our study was 64.2% in the NUH and 33.8% in the UVH, which can be explained by the high rate of patient referrals in the UVH center (47.1%, n = 32/68), while all patients were treated primarily in the NUH. A detailed evaluation shows that a relevant proportion of the empirical antibiotic regimens administered were not effective treatments against the pathogens actually present, as these exhibited corresponding resistance patterns. This shows once again that the respective characteristics of the individual cohorts have a decisive influence on the treatment of patients and that adherence to guidelines does not necessarily lead to a successful outcome.

Additionally, it is worth noting that the duration of intravenous antibiotic therapy was insufficient at NUH, while the duration of oral antibiotic therapy was excessively long, deviating from current guideline recommendations [4,10]. The literature reports that multiple pathogens are detected in approximately 10% of patients with spondylodiscitis, which significantly complicates treatment. In our study, multiple pathogens were identified in 6.7% of cases, aligning closely with literature findings [7]. Once again, a higher incidence was observed among UVH patients. This is likely attributed to the higher pre-existing disease burden among UVH patients, as noted previously. In summary, these findings underscore the importance of interdisciplinary conferences with detailed case discussions and individualized treatment strategies, especially for patients with multiple identified pathogens that require a more sophisticated treatment approach. In this context, this approach has become an integral and essential part of patient care and is now also routinely implemented in our department [24]. Interdisciplinary conferences are also well-established in other medical specialties and represent a crucial component of treatment protocols [31].

Surgical intervention for spondylodiscitis is typically indicated to obtain intraoperative bacterial samples and to address acute neurological symptoms, kyphotic deformity, and cases unresponsive to conservative management [10,11]. However, in our study, we found that more patients were treated primarily with spinal fusion of the affected segments in a UVH center than in a NUH center. Looking at the respective patient population, it is noticeable that more patients with severe pre-existing conditions, such as coexisting malignant diseases, were treated in the UVH center. Furthermore, patients at the UVH center required prolonged intensive care treatment, indicating a potentially sicker patient cohort. Another noteworthy aspect is that the UVH center received 50% of its patient cohort as transfers from other hospitals, where initial treatment failed to resolve ongoing or worsening clinical and/or neurological symptoms. Unfortunately, data regarding the rationale for surgical intervention were unavailable, thus warranting further investigation to validate these observations.

Finally, it is important to acknowledge both the limitations and strengths of our study. Firstly, we report on retrospective data, which warrants caution in interpretation. Furthermore, reliable data regarding follow-up and long-term mortality were lacking, as postoperative care was primarily conducted on an outpatient basis, and follow-up within the respective centers was infrequent. The unique strength of our study lies in the comparative analysis of two certified spine centers of differing sizes situated in distinct regions of Germany, each serving independent patient populations. This provides valuable insight into the differences between patient cohorts in different geographical regions and highlights the nuances of spondylodiscitis treatment. We were able to demonstrate in this study that there are differences in the pathogen spectrum between the two spine centers. Furthermore, we were able to demonstrate important differences in the resistance pattern of the respective pathogens, which has an important implication for the respective treatment. Our results thus emphasize that not only transregional geographical factors but also considerable regional differences within a country influence the treatment of spondylodiscitis and should, therefore, be taken into consideration. The rising incidence of this disease and the increasing number of hospitals treating this patient group underline the relevance of our data. Each hospital must consider the specific characteristics of its patient population to ensure appropriate treatment [2,8].

## 5. Conclusions

Bacterial prevalence and resistance patterns show distinct regional differences. Consequently, there is a need for standardized treatment protocols for spondylodiscitis that are adapted to regional differences. Regular re-evaluation of these protocols is essential to optimize the effectiveness of the therapy. Additionally, concomitant malignant diseases have been demonstrated to be a risk factor for spondylodiscitis.

## Figures and Tables

**Table 1:** Admission Parameters: Demographic parameters and clinical features on admission are compared in this table, showing a significant age difference and difference in hospitalization. The differences in pain prevalence and NAS are statistically significant but clinically irrelevant and may be explained by a high rate of patients with an established pain therapy at the time of presentation at a UVH. ^1^ intensive care unit; ^2^ numerical analog scale; ^3^ >37.9 °C; ^4^ normal range: <5 mg/dL.

Feature	UVH	NUH	*p*-Value
Age (years)	63.6 ± 13.4	69.2 ± 13.4	**0.020**
Female	35.3% (24)	40.3% (27)	0.550
Stay (hospital)	31.8 ± 22.6	18.9 ± 11.6	**0.002**
Stay (ICU) ^1^	8.9 ± 18.8	1.6 ± 5.2	**0.015**
**Admission**			
Acute Onset	52.9% (36)	38.5% (20)	0.115
Pain	91.9% (57)	100% (52)	**0.036**
-NAS ^2^	4.5 ± 3.0	6.6 ± 1.8	**0.001**
Neurological deficit	19.7% (13)	11.5% (6)	0.231
-Myelopathy	11.8% (8)	8.2% (4)	0.597
-Radiculopathy	7.4% (5)	4.1% (2)	
Fever ^3^	7.5% (5)	16.1 (10)	0.125
Leukocytosis	39.7% (27)	44.3% (27)	0.600
CRP-Elevation ^4^	95.6% (65)	93.9% (62)	0.668

**Table 2:** Comorbidities: This table depicts relevant comorbidities in both centers, including previous surgery and infiltration therapy. Statistically significant differences *p* < 0.05 were marked in fat writing.

Feature	UVH	NUH	*p*-Value
Previous spine surgery	17.6% (12)	17.9% (12)	0.968
Previous infiltration therapy	7.4% (5)	7.5% (5)	0.981
Diabetes	19.1% (13)	16.4% (11)	0.682
Pyogenic infection	5.9% (4)	14.9% (10)	0.085
Intravenous drug abuse	7.4% (5)	7.5% (5)	0.981
Malignancy	30.9% (21)	9.0% (6)	**0.001**
-Chemotherapy	4.5% (3)	0.0% (0)	0.078
-Port	1.5% (1)	3.0% (2)	0.551
Renal insufficiency	16.2% (11)	11.9% (8)	0.479

**Table 3:** Microbiological Group Comparison: The most common bacterial specimen were staphylococci in both centers (UVH 66.1% and NUH 65.7%), there was a trend towards more miscellaneous and tuberculosis bacteria in the UVH, however, Chi^2^ test did not reach significance, *p* = 0.13. There was a significant difference in antibiotic administration as an i.v. or oral agent. UVH patients received longer i.v. treatments.

		Microbiological Group Comparison				
		UVH		NUH		*p*-Value
Bacteria Groups	*S. Aureus*	22	37.3%	10	31.3%	0.13
	Coag. Neg. Staph.	17	28.8%	11	34.4%	
	*Enterobacterales*	4	6.8%	1	3.1%	
	*Streptococcus* sp.	1	1.7%	5	15.6%	
	*Enterococcus* sp.	4	6.8%	3	9.4%	
	*Mycobact. tuberculosis*	3	5.1%	0	0.0%	
	Miscellaneous	8	13.6%	2	6.3%	
	i.v. treatment/days	29.6 ± 22.9		9.8 ± 4.4		**<0.001**
	oral treatment/weeks	7.2 ± 5.9		12.2 ± 3.6		**<0.001**

**Table 4:** Resistances: Bacterial Resistance was high for some groups of antibiotics, especially for some of those given as a standardized empiric treatment.

	Resistances				
	UVH		NUH		*p*-Value
Penicillin	36/46	78.3%	17/24	70.8%	0.562
Isoxazolyl-Penicillin	23/46	50.0%	9/24	37.5%	0.449
Aminopenicillin	8/46	17.4%	8/24	33.3%	0.147
Cephalosporins	**4/46**	**8.7%**	**11/24**	**45.8%**	**0.001**
Carbapenems	12/46	26.1%	2/24	8.3%	0.116
Quinolons I–II	14/46	30.4%	7/24	29.2%	1.000
Quinolons III–IV	**0/46**	**0.00%**	**6/24**	**25.00%**	**0.001**
Macrolides	18/46	39.1%	10/24	41.7%	1.000
Aminoglycosides	7/46	15.2%	2/24	8.3%	0.708
Glycopeptides	4/46	8.7%	0/24	0.0%	0.291
Tetracyclines	9/46	19.6%	3/24	12.5%	0.526
Lincosamides	13/46	28.3%	9/24	37.5%	0.433
Nitroimidazoles	**1/46**	**2.2%**	**4/24**	**16.7%**	**0.044**
Sulfonamides	10/46	21.7%	4/24	16.7%	0.758
Linezolid	0/46	0.00%	1/24	4.2%	0.343
Rifampicin	4/46	8.7%	2/24	8.3%	1.000

## Data Availability

The data presented in this study are available on request from the corresponding author. The data are not publicly available due to the General Data Protection Regulation.
